# 1-[(4*S*)-4-Benzyl-2-thioxo-1,3-thia­zol­idin-3-yl]propan-1-one^1^

**DOI:** 10.1107/S1600536809030104

**Published:** 2009-08-15

**Authors:** Narendar Reddy Gade, Y. Manjula, Javed Iqbal, Peddy Vishweshwar

**Affiliations:** a Institute of Life Sciences, Hyderabad Central University Campus, Gachibowli, Hyderabad 500 046, India; b Department of Analytical Research, Discovery Research, Dr Reddy’s Laboratories Ltd, Miyapur, Hyderabad 500 049, India

## Abstract

The analysis of the title chiral auxiliary compound, C_13_H_15_NOS_2_, has enabled the determination of the absolute configuration at the benzyl-bearing ring C atom as *S*. In the crystal structure, mol­ecules aggregate into helical chains along the *b* axis *via* C—H⋯O contacts.

## Related literature

For background to the use of *N*-acyl thia­zolidinethio­nes as versatile chiral auxiliaries for asymmetric aldol reactions, see: Crimmins & Chaudhary (2000 ▶); Crimmins *et al.* (2005 ▶); Crimmins & Haley (2006 ▶); Crimmins & Dechert (2009 ▶). For the synthesis, see: McKennon & Meyer (1993 ▶); Delaunay *et al.* (1995 ▶); Lu *et al.* (2009 ▶).
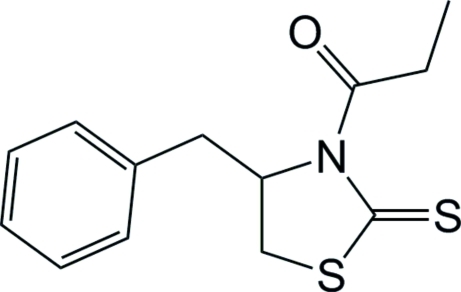


## Experimental

### 

#### Crystal data

C_13_H_15_NOS_2_
*M* *_r_* = 265.39
Monoclinic, 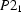

*a* = 8.850 (6) Å
*b* = 7.189 (5) Å
*c* = 10.595 (7) Å
β = 95.537 (6)°
*V* = 670.9 (8) Å^3^
*Z* = 2
Mo *K*α radiation
μ = 0.38 mm^−1^
*T* = 298 K
0.50 × 0.40 × 0.20 mm

#### Data collection

Rigaku Mercury diffractometer
Absorption correction: multi-scan (Jacobson, 1998 ▶) *T*_min_ = 0.831, *T*_max_ = 0.925
7301 measured reflections
2734 independent reflections
2361 reflections with *F* ^2^ > 2σ(*F* ^2^)
*R*_int_ = 0.038

#### Refinement

*R*[*F* ^2^ > 2σ(*F* ^2^)] = 0.041
*wR*(*F* ^2^) = 0.048
*S* = 0.86
2734 reflections
170 parameters
H-atom parameters constrained
Δρ_max_ = 0.32 e Å^−3^
Δρ_min_ = −0.35 e Å^−3^
Absolute structure: Flack (1983 ▶), 1138 Friedel pairs
Flack parameter: −0.05 (6)

### 

Data collection: *CrystalClear* (Pflugrath, 1999 ▶); cell refinement: *CrystalClear*; data reduction: *CrystalStructure* (Molecular Structure Corporation & Rigaku, 2006 ▶); program(s) used to solve structure: *SIR2004* (Burla *et al.*, 2005 ▶); program(s) used to refine structure: *CRYSTALS* (Betteridge *et al.*, 2003 ▶); molecular graphics: *X-SEED* (Barbour *et al*., 2001 ▶); software used to prepare material for publication: *CrystalStructure*.

## Supplementary Material

Crystal structure: contains datablocks global, I. DOI: 10.1107/S1600536809030104/tk2514sup1.cif

Structure factors: contains datablocks I. DOI: 10.1107/S1600536809030104/tk2514Isup2.hkl

Additional supplementary materials:  crystallographic information; 3D view; checkCIF report

## Figures and Tables

**Table 1:** Hydrogen-bond geometry (Å, °)

*D*—H⋯*A*	*D*—H	H⋯*A*	*D*⋯*A*	*D*—H⋯*A*
C9—H9⋯O1^i^	0.95	2.55	3.408 (4)	150

Symmetry code: (i) 
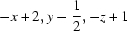
.

**Fig. 1.**
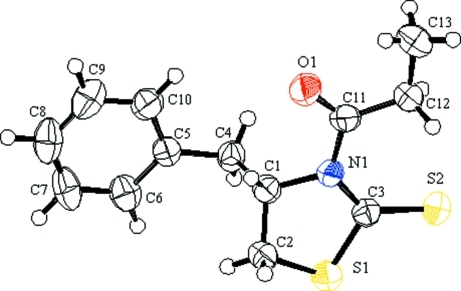
Molecular structure of (I) showing the atom numbering scheme. The displacement ellipsoids are drawn at the 50% probability level. H-atoms are shown by small circles of arbitrary radii.

**Fig. 2.**
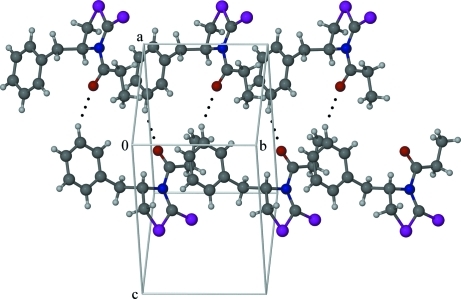
Crystal packing of (I) showing the formation of helical chains. The C-H···O contacts are shown as dashed lines.

**Fig. 3.**
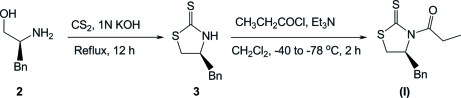
Synthesis of (I).

## supplementary crystallographic information

### Comment

*N*-Acyl thiazolidinethiones, e.g. (I), are versatile chiral auxiliaries for asymmetric aldol reactions Crimmins & Chaudhary (2000). Many complex natural products have been synthesized using these auxiliaries (Crimmins *et al.* 2005; Crimmins & Haley, 2006; Crimmins & Dechert, 2009). The synthesis of (I) starts from amino alcohol 2 which was converted to thiazolidinethione 3 by reacting with carbon disulfide followed by treatment with propionyl chloride (Fig. 3) (Crimmins & Chaudhary, 2000).

The single crystal analysis of (I), Fig. 1, allowed the determination of the absolute configuration of C1 as S. The crystal structure shows the molecules to aggregate into helical chains along the screw axis via C_9_—H_9_···O_1_ contacts (Fig. 2, Table 1).

### Experimental

To a solution of β-amino alcohol 2 (10 mmol) (McKennon & Meyer, 1993) in aqueous 1.0 N potassium hydroxide (50 ml) was added carbon disulfide (50 mmol, 3.0 ml) slowly. The reaction mixture was refluxed at 110 °C for 12 h to give the desired thiazolidinethione 3 (Delaunay *et al.* 1995). To a solution of compound 3 (0.478 mmol) in dichloromethane (DCM, 3 ml) was added triethylamine (0.956 mmol) and the temperature was maintained at -40 to -78 °C. To that mixture was added propionyl chloride (0.574 mmol) drop wise. The mixture was stirred for 1–2 h, diluted with DCM (10 ml), washed with water (2 *x* 10 ml), dried over anhydrous Na_2_SO_4_ and concentrated low vacuum to give (I) as a light-yellow solid; mp. 374–376 K (lit. mp. 374.1 K (Lu *et al.* 2009)).

Compound (I) (50 mg) was dissolved in 2:1 DCM/EtOAC (1.0 ml) and left in freezer (10 °C) until fine crystals appeared. Crystals were separated from soluton and washed with hexane and dried under vacuum.

### Refinement

The H atoms were positioned geometrically and refined in the riding model approximation with C—H = 0.95 Å, and with U_iso_(H) set to 1.2*U*_eq_(C).

### Crystal data

**Table:**

C_13_H_15_NOS_2_	*F*(000) = 280
*M**_r_* = 265.39	*D*_x_ = 1.314 Mg m^−^^3^
Monoclinic, *P*2_1_	Mo *K*α radiation, λ = 0.71070 Å
Hall symbol: P 2yb	Cell parameters from 3674 reflections
*a* = 8.850 (6) Å	θ = 2.3–27.4°
*b* = 7.189 (5) Å	µ = 0.38 mm^−^^1^
*c* = 10.595 (7) Å	*T* = 298 K
β = 95.537 (6)°	Prism, yellow
*V* = 670.9 (8) Å^3^	0.50 × 0.40 × 0.20 mm
*Z* = 2	

### Data collection

**Table:**

Rigaku Mercury diffractometer	2361 reflections with *F*^2^ > 2σ(*F*^2^)
Detector resolution: 7.31 pixels mm^-1^	*R*_int_ = 0.038
ω scans	θ_max_ = 27.4°
Absorption correction: multi-scan (Jacobson, 1998)	*h* = −11→11
*T*_min_ = 0.831, *T*_max_ = 0.925	*k* = −6→9
7301 measured reflections	*l* = −13→13
2734 independent reflections	

### Refinement

**Table:**

Refinement on *F*^2^	Chebychev polynomial *w*ith 3 parameters (Carruthers & Watkin, 1979) 10359.0000 14093.9000 3595.6900
*R*[*F*^2^ > 2σ(*F*^2^)] = 0.041	(Δ/σ)_max_ < 0.001
*wR*(*F*^2^) = 0.048	Δρ_max_ = 0.32 e Å^−^^3^
*S* = 0.86	Δρ_min_ = −0.35 e Å^−^^3^
2734 reflections	Absolute structure: Flack (1983), 1138 Friedel pairs
170 parameters	Flack parameter: −0.05 (6)
H-atom parameters constrained	

### Special details

**Table:**

Geometry. Bond distances, angles *etc*. have been calculated using the rounded fractional coordinates. All su's are estimated from the variances of the (full) variance-covariance matrix. The cell e.s.d.'s are taken into account in the estimation of distances, angles and torsion angles
Refinement. Refinement was performed using all reflections. The weighted *R*-factor (*wR*) and goodness of fit (*S*) are based on *F*^2^. *R*-factor (gt) are based on *F*. The threshold expression of *F*^2^ > 2.0 σ(*F*^2^) is used only for calculating *R*-factor (gt).

### Fractional atomic coordinates and isotropic or equivalent isotropic displacement parameters (Å^2^)

**Table:**

	*x*	*y*	*z*	*U*_iso_*/*U*_eq_	
S1	0.55104 (6)	1.08609 (10)	0.94969 (5)	0.0574 (2)	
S2	0.80540 (7)	1.31770 (10)	1.04864 (6)	0.0630 (2)	
O1	0.96079 (17)	1.0605 (2)	0.69074 (15)	0.0720 (6)	
N1	0.79082 (15)	1.1039 (2)	0.83154 (13)	0.0410 (4)	
C1	0.6994 (2)	0.9543 (2)	0.76431 (19)	0.0436 (6)	
C2	0.5366 (2)	0.9806 (2)	0.79429 (19)	0.0498 (7)	
C3	0.7324 (2)	1.1748 (2)	0.93706 (19)	0.0462 (6)	
C4	0.7616 (2)	0.7610 (2)	0.80159 (19)	0.0469 (6)	
C5	0.7183 (2)	0.6180 (2)	0.70140 (17)	0.0441 (6)	
C6	0.5872 (2)	0.5119 (2)	0.7014 (2)	0.0561 (7)	
C7	0.5469 (3)	0.3871 (3)	0.6051 (2)	0.0673 (9)	
C8	0.6379 (3)	0.3610 (3)	0.5087 (2)	0.0704 (9)	
C9	0.7667 (3)	0.4651 (3)	0.5063 (2)	0.0719 (9)	
C10	0.8074 (2)	0.5935 (3)	0.60097 (19)	0.0569 (7)	
C11	0.9289 (2)	1.1496 (2)	0.7801 (2)	0.0501 (7)	
C12	1.0320 (2)	1.3001 (3)	0.8384 (2)	0.0545 (7)	
C13	1.1629 (2)	1.3369 (4)	0.7610 (2)	0.0861 (10)	
H1	0.70330	0.96970	0.67560	0.0520*	
H6	0.52430	0.52600	0.76860	0.0670*	
H7	0.45570	0.31750	0.60530	0.0790*	
H8	0.61000	0.27260	0.44400	0.0830*	
H9	0.83030	0.44600	0.44020	0.0870*	
H10	0.89540	0.66810	0.59680	0.0690*	
H21	0.48380	1.06070	0.73390	0.0600*	
H22	0.48550	0.86440	0.79480	0.0590*	
H41	0.72130	0.72350	0.87750	0.0550*	
H42	0.86910	0.76750	0.81570	0.0560*	
H121	1.07080	1.26210	0.92110	0.0650*	
H122	0.97480	1.41110	0.84380	0.0650*	
H131	1.19300	1.46340	0.77050	0.1050*	
H132	1.24580	1.25860	0.78930	0.1050*	
H133	1.13230	1.31200	0.67430	0.1050*	

### Atomic displacement parameters (Å^2^)

**Table:**

	*U*^11^	*U*^22^	*U*^33^	*U*^12^	*U*^13^	*U*^23^
S1	0.0512 (2)	0.0621 (3)	0.0611 (3)	−0.0066 (3)	0.0167 (2)	−0.0116 (3)
S2	0.0587 (3)	0.0640 (4)	0.0664 (3)	−0.0025 (3)	0.0059 (2)	−0.0247 (3)
O1	0.0664 (9)	0.0751 (11)	0.0796 (10)	−0.0150 (9)	0.0330 (8)	−0.0265 (10)
N1	0.0401 (7)	0.0364 (8)	0.0470 (8)	−0.0001 (7)	0.0062 (6)	−0.0019 (7)
C1	0.0419 (10)	0.0430 (11)	0.0458 (10)	−0.0008 (8)	0.0035 (8)	0.0002 (8)
C2	0.0417 (10)	0.0508 (13)	0.0561 (12)	−0.0027 (9)	0.0011 (8)	−0.0019 (9)
C3	0.0427 (10)	0.0438 (11)	0.0513 (11)	0.0037 (8)	0.0008 (8)	−0.0042 (9)
C4	0.0510 (11)	0.0393 (11)	0.0487 (11)	0.0028 (8)	−0.0031 (8)	−0.0011 (8)
C5	0.0453 (10)	0.0389 (12)	0.0467 (10)	0.0008 (8)	−0.0027 (8)	0.0025 (8)
C6	0.0647 (13)	0.0460 (13)	0.0569 (12)	−0.0083 (10)	0.0017 (10)	0.0087 (9)
C7	0.0828 (17)	0.0465 (14)	0.0678 (15)	−0.0207 (11)	−0.0179 (13)	0.0093 (11)
C8	0.104 (2)	0.0511 (15)	0.0517 (13)	−0.0052 (13)	−0.0146 (13)	−0.0034 (11)
C9	0.0992 (19)	0.0603 (15)	0.0580 (14)	0.0079 (15)	0.0176 (13)	−0.0055 (12)
C10	0.0579 (11)	0.0527 (12)	0.0617 (12)	0.0015 (12)	0.0134 (9)	−0.0026 (12)
C11	0.0415 (11)	0.0480 (13)	0.0617 (12)	0.0026 (8)	0.0095 (9)	−0.0011 (9)
C12	0.0430 (10)	0.0488 (12)	0.0711 (13)	−0.0016 (10)	0.0032 (9)	−0.0073 (11)
C13	0.0543 (13)	0.086 (2)	0.121 (2)	−0.0223 (15)	0.0242 (13)	−0.0216 (19)

### Geometric parameters (Å, °)

**Table:**

S1—C2	1.806 (2)	C12—C13	1.506 (3)
S1—C3	1.744 (2)	C1—H1	0.9500
S2—C3	1.650 (2)	C2—H21	0.9500
O1—C11	1.199 (3)	C2—H22	0.9500
N1—C1	1.486 (2)	C4—H41	0.9500
N1—C3	1.374 (3)	C4—H42	0.9500
N1—C11	1.424 (2)	C6—H6	0.9500
C1—C2	1.517 (3)	C7—H7	0.9500
C1—C4	1.532 (2)	C8—H8	0.9500
C4—C5	1.501 (3)	C9—H9	0.9500
C5—C6	1.389 (3)	C10—H10	0.9500
C5—C10	1.395 (3)	C12—H121	0.9500
C6—C7	1.380 (3)	C12—H122	0.9500
C7—C8	1.373 (3)	C13—H131	0.9500
C8—C9	1.366 (4)	C13—H132	0.9500
C9—C10	1.385 (3)	C13—H133	0.9500
C11—C12	1.509 (3)		
			
C2—S1—C3	93.92 (9)	C1—C2—H21	110.00
C1—N1—C3	115.36 (14)	C1—C2—H22	111.00
C1—N1—C11	115.55 (14)	H21—C2—H22	109.00
C3—N1—C11	129.05 (14)	C1—C4—H41	109.00
N1—C1—C2	107.08 (13)	C1—C4—H42	109.00
N1—C1—C4	111.56 (15)	C5—C4—H41	108.00
C2—C1—C4	112.58 (14)	C5—C4—H42	109.00
S1—C2—C1	104.96 (13)	H41—C4—H42	109.00
S1—C3—S2	118.18 (11)	C5—C6—H6	119.00
S1—C3—N1	110.37 (12)	C7—C6—H6	120.00
S2—C3—N1	131.43 (14)	C6—C7—H7	120.00
C1—C4—C5	112.21 (15)	C8—C7—H7	119.00
C4—C5—C6	122.19 (16)	C7—C8—H8	120.00
C4—C5—C10	120.09 (16)	C9—C8—H8	121.00
C6—C5—C10	117.67 (16)	C8—C9—H9	119.00
C5—C6—C7	120.91 (19)	C10—C9—H9	120.00
C6—C7—C8	120.7 (2)	C5—C10—H10	119.00
C7—C8—C9	119.3 (2)	C9—C10—H10	120.00
C8—C9—C10	120.7 (2)	C11—C12—H121	109.00
C5—C10—C9	120.65 (18)	C11—C12—H122	109.00
O1—C11—N1	117.07 (15)	C13—C12—H121	109.00
O1—C11—C12	121.82 (17)	C13—C12—H122	109.00
N1—C11—C12	121.09 (16)	H121—C12—H122	109.00
C11—C12—C13	111.70 (18)	C12—C13—H131	109.00
N1—C1—H1	109.00	C12—C13—H132	110.00
C2—C1—H1	109.00	C12—C13—H133	109.00
C4—C1—H1	108.00	H131—C13—H132	109.00
S1—C2—H21	110.00	H131—C13—H133	110.00
S1—C2—H22	111.00	H132—C13—H133	109.00
			
C3—S1—C2—C1	22.91 (12)	C4—C1—C2—S1	93.51 (15)
C2—S1—C3—S2	171.92 (11)	N1—C1—C4—C5	−155.93 (15)
C2—S1—C3—N1	−9.64 (13)	C2—C1—C4—C5	83.7 (2)
C3—N1—C1—C2	25.15 (19)	C1—C4—C5—C6	−91.3 (2)
C3—N1—C1—C4	−98.44 (17)	C1—C4—C5—C10	86.0 (2)
C11—N1—C1—C2	−156.77 (15)	C4—C5—C6—C7	177.20 (17)
C11—N1—C1—C4	79.65 (19)	C10—C5—C6—C7	−0.1 (3)
C1—N1—C3—S1	−7.69 (18)	C4—C5—C10—C9	−178.46 (18)
C1—N1—C3—S2	170.47 (14)	C6—C5—C10—C9	−1.1 (3)
C11—N1—C3—S1	174.53 (14)	C5—C6—C7—C8	1.9 (3)
C11—N1—C3—S2	−7.3 (3)	C6—C7—C8—C9	−2.4 (3)
C1—N1—C11—O1	−2.2 (2)	C7—C8—C9—C10	1.2 (3)
C1—N1—C11—C12	179.47 (16)	C8—C9—C10—C5	0.6 (3)
C3—N1—C11—O1	175.56 (17)	O1—C11—C12—C13	7.3 (3)
C3—N1—C11—C12	−2.8 (3)	N1—C11—C12—C13	−174.42 (17)
N1—C1—C2—S1	−29.44 (15)		

### Hydrogen-bond geometry (Å, °)

**Table:**

*D*—H···*A*	*D*—H	H···*A*	*D*···*A*	*D*—H···*A*
C9—H9···O1^i^	0.95	2.55	3.408 (4)	150

Symmetry codes: (i) −*x*+2, *y*−1/2, −*z*+1.

## References

[bb1] Barbour, L. J. (2001). *J. Supramol. Chem.* **1**, 189-191.

[bb2] Betteridge, P. W., Carruthers, J. R., Cooper, R. I., Prout, K. & Watkin, D. J. (2003). *J. Appl. Cryst.* **36**, 1487.

[bb3] Burla, M. C., Caliandro, R., Camalli, M., Carrozzini, B., Cascarano, G. L., De Caro, L., Giacovazzo, C., Polidori, G. & Spagna, R. (2005). *J. Appl. Cryst.* **38**, 381–388.

[bb4] Crimmins, M. T. & Chaudhary, K. (2000). *Org. Lett.* **2**, 775–777. 10.1021/ol9913901 10754681

[bb5] Crimmins, M. T., Christie, H. S., Chaudhary, K. & Long, A. (2005). *J. Am. Chem. Soc.* **127**, 13810–13812. 10.1021/ja0549289 16201800

[bb6] Crimmins, M. T. & Dechert, A.-M. R. (2009). *Org. Lett.* **11**, 1635–1638. 10.1021/ol9003228 PMC2701212 19281219

[bb7] Crimmins, M. T. & Haley, M. W. (2006). *Org. Lett.* **8**, 4223–4225. 10.1021/ol061339e 16956192

[bb8] Delaunay, D., Toupet, L. & Le Corre, M. J. (1995). *Org. Chem.* **60**, 6604–6607.

[bb9] Flack, H. D. (1983). *Acta Cryst.* A**39**, 876–881.

[bb10] Jacobson, R. (1998). Private communication to the Rigaku Corporation, Tokyo, Japan.

[bb11] Lu, C., Nie, J., Yang, G. & Chen, Z. (2009). *Can. J. Chem.* **87**, 30–32.

[bb12] McKennon, M. J. & Meyer, A. I. (1993). *J. Org. Chem.* **58**, 3568–3571.

[bb13] Molecular Structure Corporation & Rigaku (2006). *CrystalStructure* MSC, The Woodlands, Texas, USA, and Rigaku Corporation, Tokyo, Japan.

[bb14] Pflugrath, J. W. (1999). *Acta Cryst.* D**55**, 1718–1725. 10.1107/s090744499900935x 10531521

